# Lack of Genetic Structure and Female-Specific Effect of Dispersal Barriers in a Rabies Vector, the Striped Skunk (*Mephitis mephitis*)

**DOI:** 10.1371/journal.pone.0049736

**Published:** 2012-11-14

**Authors:** Benoit Talbot, Dany Garant, Sébastien Rioux Paquette, Julien Mainguy, Fanie Pelletier

**Affiliations:** 1 Département de biologie, Université de Sherbrooke, Sherbrooke, Québec, Canada; 2 Canada Research Chair in Evolutionary Demography and Conservation, Université de Sherbrooke, Sherbrooke, Québec, Canada; 3 Service de la biodiversité et des maladies de la faune, Ministère des Ressources naturelles et de la Faune, Québec, Québec, Canada; Monash University, Australia

## Abstract

Evaluating the permeability of potential barriers to movement, dispersal and gene exchanges can help describe spreading patterns of wildlife diseases. Here, we used landscape genetics methods to assess the genetic structure of the striped skunk (*Mephitis mephitis*), which is a frequent vector of rabies, a lethal zoonosis of great concern for public health. Our main objective was to identify landscape elements shaping the genetic structure of this species in Southern Québec, Canada, in an area where the raccoon rabies variant has been detected. We hypothesised that geographic distance and landscape barriers, such as highways and major rivers, would modulate genetic structure. We genotyped a total of 289 individuals sampled across a large area (22,000 km^2^) at nice microsatellite loci. Genetic structure analyses identified a single genetic cluster in the study area. Major rivers and highways, however, influenced the genetic relatedness among sampled individuals. Sex-specific analyses revealed that rivers significantly limited dispersal only for females while highways only had marginal effects. Rivers and highways did not significantly affect male dispersal. These results support the contention that female skunks are more philopatric than males. Overall, our results suggest that the effects of major rivers and highways on dispersal are sex-specific and rather weak and are thus unlikely to prevent the spread of rabies within and among striped skunk populations.

## Introduction

Studies investigating patterns of gene flow and dispersal among and within populations can help understand evolution on ecological time scales (reviewed in [1], [2]). Gene flow and the resulting genetic structure of populations are typically shaped by landscape heterogeneity (reviewed in [3]–[5]). For example, in addition to geographical distance, landscape barriers to animal movement, whether of natural or anthropogenic origin, have been shown to often cause a reduction in gene flow among populations [6]–[9]. Landscape genetics approaches have been developed to detect reductions in gene flow caused by these elements at different spatial scales [10]–[12]. They offer a robust framework to improve our understanding of the evolutionary processes affecting a species in a given area, as well as insights into habitat preferences, sex-biased dispersal and community association [13]–[16].

Recently, landscape genetic approaches have been increasingly used for applied purposes [17]–[20], especially in the study and management of wildlife diseases [21]–[23]. As several diseases are propagated by wild animals, identifying landscape elements that can limit or prevent gene flow can be used to better target intervention actions [13], [24], [25] or to make predictions about the most probable paths for disease spread [22], [26]. For instance, in order to prevent further raccoon rabies in Ontario, the Ontario Ministry of Natural Resources and its partners have designated oral rabies vaccination (ORV) zones on the New York/Ontario international border along the St. Lawrence River [27] and along the Niagara River [28], where raccoons tested positive for rabies were euthanized. The efficiency of these ORV zones can be improved if integrated along landscape features that naturally limit disease vector dispersal. Assessing the permeability of landscape elements is therefore important for such applied purposes.

Landscape features and geographic distance may influence gene flow differently for each vector species depending on their ecology, their life-history traits, and the scale at which the system is studied. Generally, geographic distance has a stronger effect on gene flow in species with a limited dispersal potential compared to those able to disperse over great distances (e.g., [29]). For example, pairwise geographic distance explained more of the observed genetic variation between sampling sites for the American mink (8%, *Neovison vison*; [30]), than for American martens (0.3%, *Martes americana*; [31]), the latter exhibiting generally greater average dispersal distances than the former [32]. Barriers to gene flow will also have differential permeability depending on the capacity of a species to cross or bypass them and it is thus important to characterize broad and fine scale genetic structure of each specific disease vector independently.

A fine-scale genetic structure can be detected using patterns of genetic relatedness among individuals (e.g., [33]–[35]). Such fine-scale structure analyses are helpful to assess population kin structure [36] and have been shown to be linked with the rate of inbreeding (e.g. [37]). In addition, it allows detecting landscape features that may have sex-specific effects. Male-biased dispersal and female philopatry have been suggested as possible mechanisms for inbreeding avoidance in mammals because the direction of dispersal bias is tightly linked to the mating system (reviewed in [38]) and most mammals are polygynous with mate defence (reviewed in [39]). In carnivores for instance, evidence shows that most species exhibit male-biased dispersal, as previously documented in American black bears (*Ursus americanus*, [40]), wolverines (*Gulo gulo*, [41]) and American mink [30].

The main objective of this study was to assess the extent of genetic structuring in striped skunks (*Mephitis mephitis*: Mephitidae) in an area where cases of the raccoon rabies variant were found in skunks and raccoons [42] in order to evaluate the permeability of landscape barriers (highway and rivers) to gene flow. The striped skunk is a medium-sized nocturnal North American mesocarnivore [43], which is often abundant in cities [44], [45] and is also abundant in farmsteads and cropfield edges where prey items are abundant [46], [47]. Home range size in this species is generally around 3 km^2^ [48]. It has been suggested that striped skunks in their native North American range originated from a common ancestor in the Texas-Mexico region and expanded north into several clades, one of which is present in northeastern North America, where our study took place [49]. Striped skunks are known to host several strains of rabies, a zoonotic disease of great concern for public health in North America [50], including the raccoon (*Procyon lotor*) variant of the rabies virus [51]. This rabies variant has been involved in one of the most important outbreaks of wildlife rabies in the United States [52]. In the last decade, rabid skunks have been found in Ontario and Québec, including in our study area [42]. Despite the close proximity of striped skunks to human infrastructure and the zoonotic risks involved, information is still lacking about the spatial organization and movement of this species. Identifying the determinants of the genetic structure in this species could provide insights into the potential of disease spread among striped skunk populations, which could then be used to predict the extent and most probable locations of epidemiologic outbreaks.

Specifically, we tested the hypothesis that geographic distance and landscape barriers (rivers and highways) are drivers of genetic structure in our study area. The purpose of our study is to determine if some landscape barriers can be targeted to improve efficiency of ORV zones. We hypothesized that isolation by distance (IBD) in the population would be detected, because daily movement rates of skunks are thought to be small (e.g., 1.25 km, [53]) and dispersal is typically <3 km ([54], but see [55] for a documented distance of 119 km in a marked individual). In comparison with average distances traveled by similar-size mesocarnivores, skunks can be regarded as relatively sedentary [56]. We also predicted that large rivers (physical barriers) and roads (behavioural avoidance and sources of mortality) would limit gene flow in skunks, as previously reported in other mesocarnivores (raccoons [24], [35] and badgers, *Meles meles* [10]). Finally, we hypothesized that males are more likely to disperse than females and thus, that sex-biased dispersal could be detected indirectly through differences in observed patterns of pairwise genetic relatedness in each sex. This study uses current genetic methods to provide useful information on the dispersal behavior in a lesser known rabies vector, the striped skunk.

## Materials and Methods

### Ethics Statement

All field operations were conducted by the personnel of the Ministère des Ressources Naturelles et de la Faune (MRNF) Québec government agency and its partners in a disease management perspective in areas of Québec where rabies was detected. Animal trapping and handling methods complied with the Agreement on International Humane Trapping Standards (Government of Canada, 1998). Biopsy sampling protocol was approved by the Canadian Council on Animal Care (protocol numbers are CPA-FAUNE 2009–12 and CPA-FAUNE 2010–29). Captured live animals were anesthetised by trained professionals under the supervision of a veterinarian.

### Study Area and Sample Collection

**Figure 1:**
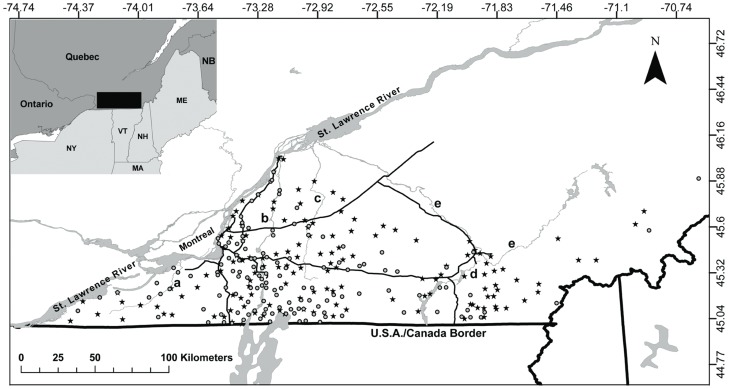
Map of the study area in Southern Québec, Canada. The location of sampled striped skunk (*Mephitis mephitis*) in this study is shown by a black star for 2009 (*n* = 148) sampling year and a grey circle for 2010 (*n* = 141) sampling year. Grey shapes are major bodies of water such as rivers and lakes in and around the study area. Thin black lines represent major highways found in the study area. St Lawrence River and the 5 selected rivers for our analyses (a: Châteauguay, b: Richelieu, c: Yamaska, d: Magog, e: Saint-François) are identified on the map. The city of Montréal is also identified.

Skunks were sampled over two years in a fragmented agricultural landscape in Southern Québec, Canada (45° 23′ N, 72° 43′ W), in an area of approximately 22 000 km^2^ (Figure 1). Tissue samples used in this study were collected by the MRNF and its partner agencies in 2009 and 2010 during the surveillance and control activities conducted against the raccoon variant of rabies, such as the recovery of roadkills for rabies testing and vaccination operations [42]. Each time an animal was recovered, its location was recorded using a handheld global positioning system. A skin biopsy was collected from the ear with a 2-mm punch for subsequent genetic analyses. Samples were stored in 95% ethanol until DNA extraction. Because many skunks were sampled over small areas due to control operations conducted for a specific zone (≈ 20 individuals/100 km^2^ zones), a random subset of individuals (up to 3 individuals/25 km^2^) was selected for each year to generate a balanced sample over the study area (Figure 1).

### Landscape Characterization

Major rivers and highways were mapped using aerial photographs of Southern Québec provided by the MRNF (Figure 1) with ArcGIS 9.2 (Environmental Systems Research Institute, Redlands, CA, USA). Major rivers were determined based on their mean water flow (m/sec) [57]. We selected rivers with a water flow greater than 0.6 m/s because they are known to maintain substantial discharge throughout the year, potentially limiting gene flow. Based on this characteristic, four rivers were retained for our analyses (rivers a, c, d and e in Figure 1). The Richelieu River (river b, Figure 1) was also considered because it was previously found to affect the genetic relatedness structure of female raccoons in this area [35]. Lakes in the course of each river were also taken into account. Highways were selected based on the definition provided by the Ministère des Transports du Québec, which defines major highways as being roads with high speed limit (100 km/h) and without crossroads (Figure 1; note that the highways considered here have no veterinary fences). To determine the number of highways and rivers separating each pair of individuals, we calculated how many times the shortest geographical distance between each pair of samples intersected with a section of a river or with a section of a highway using Hawth’s Tools (an extension of ArcGIS 9).

### DNA Extraction and Genetic Analyses

DNA extraction was conducted using a salting out protocol as described in Chambers and Garant [58]. Microsatellite polymorphism was then analyzed at the following nine polymorphic loci developed specifically for striped skunks: Meph42-15, Meph22-16, Meph22-70, Meph42-73, Meme84, Meph22-14, Meme15, Meme75, Meph22-19 ([59], [60]; see Table S1 for details). DNA amplification was performed using GeneAmp System 9700 thermocyclers (Applied Biosystems, Foster, CA, USA). Further details on polymerase chain reaction (PCR) reagent volume, concentrations and amplification conditions for each locus are supplied in Tables S2 and S3. Genotyping was performed using an AB 3130 DNA sequencer (Applied Biosystems). For each sample, a volume of 1 µL of PCR product was added to 8.9 µL of Hi-Di Formamide and 0.1 µL of Genescan Liz 600 (Applied Biosystems). Allele size was scored using GeneMapper 4.0 (Applied Biosystems).

Molecular sexing was conducted using a protocol modified from Shaw et al. [61]. Amplification was performed in a 25 µl solution containing final concentrations of 0.3 µM of each primer (LGL331 and LGL335), 0.4 mM of nucleotide triphosphate, 0.9 mM of MgCl_2_, 2.5 µg of bovine serum albumin, 1x PCR buffer (50 mM KCl, 20 mM Tris HCl, pH 8.4), 1 unit of *Taq* polymerase (Life Technologies) and 25 ng of DNA. PCR products were visualized after a 10 minutes electrophoresis migration at 300 volts on 1% agarose gels in a sodium borate buffer (10 mM NaOH pH 8.5 adjusted with H_3_BO_3_) with ethidium bromide. A 100-bp DNA ladder (Life technologies) was used each time to standardize the migration.

Despite the high reliability of the method, sex could not be determined for 19% of the selected samples, possibly due to degraded DNA (some animals died several days before their recovery). Consequently, we had a smaller sample size of known-sex individuals (104 females and 129 males).

### Microsatellite Polymorphism

We tested all loci for departure from Hardy-Weinberg equilibrium and calculated inbreeding coefficient (*F*_is_) for each locus using the software Genepop 4.0 [62]. We also tested for linkage disequilibrium using the software Fstat 2.9.3.2 [63]. Finally, we used Cervus 3.0 [64], [65] to calculate the number of alleles, observed and expected heterozygosities, and to test for the presence of null alleles. Significance of these tests was assessed after Bonferroni correction [66].

### Population Genetic Structure

To estimate the most likely number of genetic clusters in the sample, we used the Bayesian clustering software Structure 2.3.3 [67]. We performed the analyses using a model with admixture, separate admixture coefficients (α) for each genetic cluster, allele frequencies correlated (using allele frequency prior (λ) = 1.0) among genetic clusters, and without using prior information on sampling location. For each value of K (K being the number of genetic clusters considered: from 1 to 5), we ran 10 independent models with 500 000 iterations, plus a burn-in period of 100 000 iterations. Means of the ln-probabilities of all independent runs for a given K were then calculated. We ran these analyses for all individuals together and then for each sex separately.

### Relatedness Genetic Structure

To estimate the effects of landscape features on pairwise genetic relatedness among individuals, we used Multiple Regressions on distance Matrices [68]. This method is an extension of Mantel tests [69] and partial Mantel tests [70] with more than two explanatory matrices, and we used permutations to calculate type 1 error probability values [71]. These analyses account for pseudo-replication in pairwise individual relationships and thus allow regression on distance matrices. We performed Multiple Regressions on distance Matrices analyses using the package Ecodist V1.2.2 [72] in R 2.11.1 (R Foundation for Statistical Computing, Vienna, Austria), with 10 000 permutations and Pearson’s correlation coefficient. Genetic distances among individuals were calculated using the pairwise genetic relatedness (r_xy_) estimator of Wang [73], with the program Spagedi V1.3 [74]. Relatedness estimators are measures of genetic similarity between pairs of individuals. In the analyses, we used 1- r_xy_ to generate a pairwise genetic distance metric, as in Côté et al. [35]. Geographic distance (km) between each pair of individuals was measured using Hawth’s Tools to account for a possible pattern of IBD in our study system. Matrices of geographic distances (km), number of major rivers, number of highways and year of sampling (to assess temporal stability) between each pair of individuals were used both in separate models (univariate analyses) and in a single model where total variance explained was parted among the four factors (multivariate analyses). We also ran the analyses for each sex separately to test for differences between sexes. In each case, we applied jackknife resampling, removing one individual from each replicate, to estimate the standard error of model coefficients following Efron and Tibshirani [75]. This allowed the computation of 95% confidence intervals. In addition, it allowed testing whether slopes estimated for each sex in multivariate models were significantly different or not, using Student’s *t* test.

## Results

### Microsatellite Polymorphism

A total of 289 skunks were genotyped at nine microsatellites. Number of alleles identified per locus varied between 7.0 and 19.0 (mean = 11.9) and expected heterozygosity varied between 0.615 and 0.899 (mean = 0.807; see Table 1). We found neither a significant heterozygote deficit nor excess in our sample, and no evidence suggesting the presence of null alleles (lower than 5%, Table 1) or linkage disequilibrium (results not shown).

**Table 1:** Number of alleles (A), observed (H_O_) and expected (H_E_) heterozygosity, inbreeding coefficient (*F*_is_), and the probability for null alleles for the nine microsatellite loci of the striped skunk (*Mephitis mephitis*) used in our study in Southern Québec, Canada (N = 289) in 2009 and 2010.

Locus	A	H_O_	H_E_	*F*_is_	Null alleles
Meph42–15	7	0.578	0.615	0.060	0.033
Meph22–16	10	0.752	0.780	0.036	0.015
Meph22–70	19	0.877	0.899	0.024	0.012
Meph42–73	13	0.801	0.829	0.034	0.016
Meme84	12	0.815	0.852	0.043	0.021
Meph22–14	15	0.869	0.852	−0.019	−0.011
Meme15	9	0.751	0.765	0.018	0.012
Meme75	13	0.876	0.871	−0.005	−0.004
Meph22–19	9	0.782	0.804	0.028	0.015
Overall	11.9	0.789	0.807	0.024	N/A

### Population Genetic Structure

**Figure 2:**
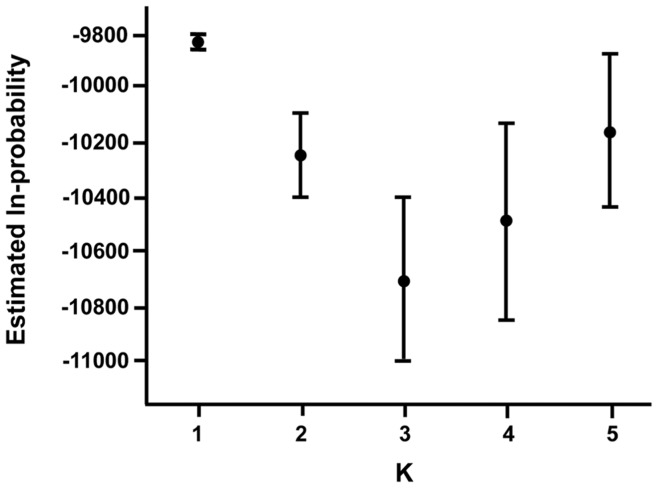
Number of genetic clusters observed in the study area. Mean and standard deviation of estimated ln-probabilities of data are presented for genetic clusters K = 1 to 5, calculated with the Structure software for striped skunks (*Mephitis mephitis*) in the study area in Southern Québec, Canada, in 2009 and 2010.

The results obtained with Structure indicated that the most likely number of genetic clusters was K = 1 (Figure 2), suggesting that striped skunks found in our study area were not subdivided in many distinct clusters. When sexes were analysed separately, Structure also provided evidence for K = 1 (results not shown), suggesting that the genetic structure within each sex was similar to that detected using the whole sample.

### Relatedness Genetic Structure

Univariate regression analyses showed significant relationships between geographic distance, number of major rivers, number of highways and genetic distance among individuals (models 1, 2 and 3 in Table 2). However, multivariate regression analysis only recovered a significant relationship for rivers and a marginally non-significant relationship for highways, whereas the effect of geographic distance was no longer significant (model 5 in Table 2). This loss of significance observed here is likely due to collinearity between geographic distance and barrier variables (*r* = 0.705 with number of rivers and *r* = 0.451 with number of highways). No significant effect of the year of sampling was detected (models 4 and 5 in Table 2).

**Table 2:** Results of univariate and multivariate regression analyses of genetic distance matrices with the MRM [68], using 10 000 permutations, for striped skunks (*Mephitis mephitis*) originating from Southern Québec, Canada, in 2009 and 2010.

Type of test	Model	Explanatory variable(s)	Slope (95% CI)	*P*
**Univariate**	1	Geographic distance	0.0002 (0.0001,0.0003)	0.026
	2	Number of rivers	0.0089 (0.0041,0.0137)	< 0.001
	3	Highways	0.0069 (0.0023,0.0115)	0.002
	4	Year	−0.0002 (−0.0045,0.0041)	0.88
**Multivariate**	5	Geographic distance	−2×10^−5^ (−0.0002,0.0002)	0.83
		Rivers	0.0073 (0.0008,0,0138)	0.007
		Highways	0.0051 (−0.0001,0.0103)	0.052
		Year	−0.0008 (−0.0052,0.0036)	0.59

In the analyses performed for each sex separately, differences in the association between pairwise genetic relatedness and landscape features were observed between sexes. Univariate regression analyses (models 1, 2 and 3 in Table 3) again showed positive and significant relationships between geographic distance, number of major rivers, number of highways and genetic distance for females, but showed only non-significant relationships for males. In females, multivariate regression analyses also showed a significant and positive effect of major rivers and a marginally non-significant and positive effect of highways on genetic distance among individuals, but no effect of geographic distance (model 5 in Table 3). In males, multivariate regression analyses also showed that none of the landscape features considered constituted a significant predictor of the genetic distance among males (model 5 in Table 3). Despite a 20-fold difference, slopes were not statistically different between sexes (Table 3), which is a reflection of the large standard errors for males. Again, no significant effect of the year of sampling was detected (models 4 and 5 in Table 3).

**Table 3:** Results of univariate and multivariate regressions analyses of genetic distance matrices with the MRM [68], using 10 000 permutations for each sex separately in striped skunks (*Mephitis mephitis*) originating from Southern Québec, Canada, in 2009 and 2010 (*P* values for slopes between sexes were calculated using Student’s *t* test).

Sex			Females		Males		*P* of slope difference between sexes
Type of test	Model	Explanatory variable	Slope (95% CI)	*P*	Slope (95% CI)	*P*	
**Univariate**	1	Geographic distance	0.0003 (0.0001,0.0005)	0.011	0.0001 (−0.0001,0.0003)	0.27	–
	2	Number of rivers	0.0154 (0.0070,0.0238)	< 0.001	0.0048 (−0.0043,0.0139)	0.26	–
	3	Number of highways	0.0129 (0.0038,0.0220)	0.001	0.0036 (−0.0041,0.0113)	0.34	–
	4	Year	0.0059 (−0.0113,0.0231)	0.46	0.0016 (−0.0105,0.0137)	0.73	–
**Multivariate**	5	Geographic distance	−0.0001 (−0.0005,0.0003)	0.56	0.0001 (−0.0002,0.0004)	0.55	0.42
		Number of rivers	0.0138 (0.0025,0.0250)	0.005	0.0007 (−0.0109,0.0123)	0.89	0.11
		Number of highways	0.0088 (−0.0017,0.0193)	0.058	0.0017 (−0.0068,0.0102)	0.70	0.30
		Year	0.0053 (−0.0117,0.0223)	0.51	0.0018 (−0.0102,0.0138)	0.70	0.74

## Discussion

The goal of our study was to investigate the population genetic structure of the striped skunk in a heterogeneous landscape in Southern Québec, Canada. We hypothesised that geographic distance and landscape barriers, such as highways and major rivers, would create genetic structure for this species in our study area, and that structure would be greater for females than for males. Our results suggest the presence of a single genetic cluster at the spatial scale studied (22 000 km^2^). Using a pairwise genetic relatedness estimator at the individual level, however, we found that landscape features, such as highways and major rivers, influenced the structure of genetic relatedness between individuals and especially females. Our results thus support the contention that males disperse more than females in this species and would likely be more active vectors of rabies propagation.

### Population Genetic Structure

Analyses performed with Structure suggested a single genetic cluster, indicating a low level of genetic differentiation over our study area. Similar results were obtained in two studies in the USA on this species. No genetic structure was found among groups of skunks in a 61.6 km^2^ study area in Texas [76], and a more recent study also failed to find significant genetic population structure over a larger area encompassing parts of North Dakota to Oklahoma (1,250 km^2^, [77]). Altogether, our results and previous evidence suggest a relatively high level of gene flow among groups of skunks which prevents population differentiation over a large scale. It should also be noted that a lack of genetic structure was reported for raccoon individuals sampled in southern Québec (over the same area as in our study – see [35]), suggesting that admixture among groups of individuals might be a common pattern for these two mesocarnivores in this area.

### Relatedness Genetic Structure

At first, univariate regression analyses assessing the effect of geographic distance on pairwise genetic relatedness revealed significant patterns of isolation by distance (lower relatedness among individuals located further apart). However, when major rivers and highways were taken into account in multivariate analyses, the IBD signal was no longer detected. In cases where past or current barriers to gene flow are present in a study area, a relationship between geographic distance and genetic distance might potentially reflect the effect of barriers to gene flow rather than the simple effect of geographic distance (e.g., [58], [78]).

We also found that major rivers and, to some extent, highways affected genetic relatedness of striped skunk individuals sampled in Southern Québec and that this effect was detected in females but not in males. More specifically, female individuals located on opposite sides of these landscape elements were generally less related than individuals located on the same side. A similar effect was detected for raccoons separated by the Richelieu River in this area (river b on Figure 1; [35]). Individual raccoons that were separated by the Richelieu River were more distant genetically than individuals located on the same side and this effect was detected in females, but not in males [35]. Our results are also similar to those reported in a study of a badger population structure in England where the presence of a large river and motorway were shown to influence patterns of relatedness among individuals [10]. In this previous study, however, the possibly differential effect on both sexes was not considered.

Previous evidence, obtained from studies using live trapping and radio-tracking methods, suggested a greater dispersal in male than in female striped skunks ([55], but see also [54], [56]). Furthermore, in their study of skunks in Texas, Hansen et al. [76] reported that males within a given sampled group had lower average genetic similarity than females of the same group, also suggesting greater dispersal for males. Here, we found that genetic distance among males was not affected by the presence of highways and major rivers, also suggesting a stronger dispersal in males. Stronger male dispersal and female philopatry are thus also consistent with the patterns observed in the majority of mammals (reviewed in [38], [39]).

Studies of habitat selection have shown that skunks are often found near roads. For instance, Frey and Conover [48] showed that skunks tend to include roadsides in their home range more often than by chance and Hwang et al. [79] found that den sites were often closer to roads than to random sites. These studies suggest that striped skunks may often attempt to cross roads. In contrast to this presumption, our results suggest a marginally positive effect of the number of roads on female genetic distance. One possible explanation for these seemingly opposing results is that the study by Frey and Conover [48] was conducted in an area were most roads are secondary roads (Bear River Migratory Bird Refuge, United States) while here only highways (higher driving speed limit and more driving lanes) were considered in our analysis. Thus, while skunks could use areas along small roads to increase their foraging opportunities, they probably avoid crossing large highways. While this still remains to be properly tested, we suggest the existence of a threshold in the permeability of the roads under which striped skunks may attempt to cross them (e.g., width, noise, traffic flow).

Our study is the first to characterize fine-scale genetic differentiation and population structure in striped skunks over a large-spatial scale. Although we found no evidence of strong genetic structuring over the scale of our study area, our results indicate reduced dispersal across rivers and highways and suggest that the effects of these barriers mostly affect females. Our study also provides evidence for male-biased dispersal supporting the hypothesis of sex differences in dispersal in this species and thus, our results offer additional insights into the dispersal behaviour of the striped skunk.

From a disease management perspective, our results indicate that major roads and rivers are insufficient to halt or slow the spread of rabies in striped skunks. Using oral rabies vaccination over the landscape, regardless of the presence of barriers, is thus essential in an attempt to eradicate rabies.

## Supporting Information

Table S1 Marker name, GenBank accession number, primer sequences, fluorescence dye used (on the forward primer), repetition pattern and lengths of alleles for the nine microsatellite loci used in this study in Southern Québec, Canada, in 2009 and 2010 (^1^Dragoo et al. [59] and ^2^Munguia-Vega et al. [60]).
(DOC)

Table S2 Polymerase chain reaction reagent volume and concentrations (final volume of 10 µL per sample with 10 ng of DNA) for the nine microsatellite loci used in this study in Southern Québec, Canada, in 2009 and 2010 (Modified from Dragoo et al. [59] and Munguia-Vega et al. [60]).
(DOC)

Table S3 Amplification conditions for the nine microsatellite loci used in this study in Southern Québec, Canada, in 2009 and 2010 (Modified from Dragoo et al. [59] and Munguia-Vega et al. [60]).
(DOC)
