# Supplementary material for: Lack of Genetic Structure and Female-Specific Effect of Dispersal Barriers in a Rabies Vector, the Striped Skunk (Mephitis mephitis)
Source: PLoS One. 2012 Nov 14;7(11):e49736. doi: 10.1371/journal.pone.0049736 (PMC3498222; doi:10.1371/journal.pone.0049736)
Supplement: Table S1 — Marker name, GenBank accession number, primer sequences, fluorescence dye used (on the forward primer), repetition pattern and lengths of alleles for the nine microsatellite loci used in this study in Southern Québec, Canada, in 2009 and 2010 (1Dragoo et al. [59] and 2Munguia-Vega et al. [60]). (DOC) [file pone.0049736.s001.doc]

**Table S1.** Marker name, GenBank accession number, primer sequences, fluorescence dye used (on the forward primer), repetition pattern and lengths of alleles for the nine microsatellite loci used in this study in Southern Québec, Canada, in 2009 and 2010 (^1^Dragoo et al. [59] and ^2^Munguia-Vega et al. [60]).

| **Marker Name** | **# GenBank** | **Primer Sequences** | **Dye** | **Repetition Pattern** | **Lenght of alleles (bp)** |
| --- | --- | --- | --- | --- | --- |
| Meph42–15^1^ | EU623429 | F: CATTTGAACGATATTCTCCCCATCC | PET | (CA)^15^ | 175–195 |
|  |  | R: CTGACGTTTCTCAGCTGTTTAGGAAG |  |  |  |
| Meph22–16^1^ | EU623424 | F: GATCCCCCAAAACACAAAAACTATG | FAM | (GT)^17^ | 260–278 |
|  |  | R: GCTGGATAGCGCTGGCATG |  |  |  |
| Meph22–70^1^ | EU623427 | F: CAGATGCATCAGCAACGATTC | VIC | (CA)^24^ | 183–231 |
|  |  | R: GAGTGTTGCATTCAGCCTGTG |  |  |  |
| Meph42–73^1^ | EU623432 | F: AAAGGACAATCCCACAGGTCT | FAM | (CA)^14^ | 142–166 |
|  |  | R: TGGACATGGAATTCTGGTTG |  |  |  |
| Meme84^2^ | GQ453416 | F: GCAAAGGATATATTTGATAAGGGATT | NED | (CA)^15^ | 149–176 |
|  |  | R: AATGGCTTTGTTTCCAGCAG |  |  |  |
| Meph22–14^1^ | EU623423 | F: CTTTTGGGTCATTAGTGCATTTATG | VIC | (GT)^24^ | 241–269 |
|  |  | R: GGAAAGAGGAAAGAAAACCCATG |  |  |  |
| Meme15^2^ | GQ453410 | F: CCAGGAAAGCCACTGAAAGA | VIC | (GT)^20^ | 153–169 |
|  |  | R: TCCTTACACGCTCCTTCTGC |  |  |  |
| Meme75^2^ | GQ453413 | F: GTGTAGCTCTTCAGAGATGGATAGG | FAM | (GT)^22^ | 137–162 |
|  |  | R: TTCCAGGATGAACCAGGATG |  |  |  |
| Meph22–19^1^ | EU623425 | F: CAGGCATCTTTGTAGGGAAGG | VIC | (CA)^22^ | 309–331 |
|  |  | R: AACATTCCCGACCAGCAAC |  |  |  |
