# Supplementary material for: Lack of Genetic Structure and Female-Specific Effect of Dispersal Barriers in a Rabies Vector, the Striped Skunk (Mephitis mephitis)
Source: PLoS One. 2012 Nov 14;7(11):e49736. doi: 10.1371/journal.pone.0049736 (PMC3498222; doi:10.1371/journal.pone.0049736)
Supplement: Table S2 — Polymerase chain reaction reagent volume and concentrations (final volume of 10 µL per sample with 10 ng of DNA) for the nine microsatellite loci used in this study in Southern Québec, Canada, in 2009 and 2010 (Modified from Dragoo et al. [59] and Munguia-Vega et al. [60]). (DOC) [file pone.0049736.s002.doc]

**Table S2.** Polymerase chain reaction reagent volume and concentrations (final volume of 10 µL per sample with 10 ng of DNA) for the nine microsatellite loci used in this study in Southern Québec, Canada, in 2009 and 2010 (Modified from Dragoo et al. [59] and Munguia-Vega et al. [60]).

|  | **Microsatellite loci** | | | | |
| --- | --- | --- | --- | --- | --- |
| **Reagent** | **Meme75**  **Meph42-15**  **Meph22-70** | **Meph22-16**  **Meph42-73** | **Meme84**  **Meme15** | **Meph22-14** | **Meph22-19** |
| PCR buffer* | 1X | 1X | 1X | 1X | 1X |
| MgCl2 (mM) | 2.5 | 2.5 | 3 | 2.5 | 3 |
| dNTPs (mM) | 0.08 | 0.08 | 0.08 | 0.14 | 0.08 |
| BSA (µg) | 0.4 | 0.4 | 0.4 | 0.4 | 0.4 |
| Forward primer (µM) | 0.03 | 0.02 | 0.03 | 0.03 | 0.02 |
| Reverse primer (µM) | 0.03 | 0.02 | 0.03 | 0.03 | 0.02 |
| *Taq* polymerase (unit) | 0.1 | 0.1 | 0.1 | 0.1 | 0.1 |

*1X PCR buffer 10 mM of Tris-HCl pH 9.0, 5 mM KCl, Triton X-100 1% and Tween-20 1%
