# Supplementary material for: Lack of Genetic Structure and Female-Specific Effect of Dispersal Barriers in a Rabies Vector, the Striped Skunk (Mephitis mephitis)
Source: PLoS One. 2012 Nov 14;7(11):e49736. doi: 10.1371/journal.pone.0049736 (PMC3498222; doi:10.1371/journal.pone.0049736)
Supplement: Table S3 — Amplification conditions for the nine microsatellite loci used in this study in Southern Québec, Canada, in 2009 and 2010 (Modified from Dragoo et al. [59] and Munguia-Vega et al. [60]). (DOC) [file pone.0049736.s003.doc]

**Table S3. Amplification conditions for the nine microsatellite loci used in this study in Southern Québec, Canada, in 2009 and 2010 (Modified from Dragoo et al. [59] and Munguia-Vega et al. [60]).**

| **PCR** | **Amplification conditions** | **Loci** |
| --- | --- | --- |
| 1 | 94 °C 5 min, 40 cycles at 94 °C 30 s, 65°C 45 s, 72 °C 45 s, and 72 °C 10 min | Meph42-15, Meph22-16 |
| 2 | 94 °C 5 min, 30 cycles at 94 °C 30 s, 61 °C 45 s, 72 °C 45 s, and 72 °C 10 min | Meph22-70 |
| 3 | 94 °C 5 min, 40 cycles at 94 °C 30 s, 61 °C 45 s, 72 °C 45 s, and 72 °C 10 min | Meph42-73, Meme84, Meph22-14, Meme15 |
| 4 | 94 °C 5 min, 40 cycles at 94 °C 30 s, 59 °C 30 s, 72 °C 30 s, and 72 °C 7 min | Meme75, Meph22-19 |
